# Cytotoxic function of CD8+ T lymphocytes isolated from patients with acute severe cerebral infarction: an assessment of stroke-induced immunosuppression

**DOI:** 10.1186/1471-2172-14-1

**Published:** 2013-01-03

**Authors:** Gang Li, Xin Wang, Li-hong Huang, Yue Wang, Jun-jie Hao, Xia Ge, Xiao-yun Xu

**Affiliations:** 1 Department of Neurology, East Hospital, Tongji University School of Medicine, Shanghai, 200120, China; 2 Department of Neurology, Zhongshan Hospital, Fudan University, Shanghai 200032, China; 3 Department of Neurology, Central Hospital of Shanghai Zhabei District, Shanghai, 200070, China

**Keywords:** CD8+ T lymphocytes, Cerebral infarction, Cytotoxic function

## Abstract

**Background:**

There is increasing evidence on complex interaction between the nervous and immune systems in patients with cerebral infarction. This study was conducted to evaluate cytotoxic function of CD8^+^ T lymphocytes isolated from patients with acute severe cerebral infarction. In order to determine role of immune system in stroke, peripheral blood mononuclear cells (PBMCs) were taken and cytotoxic function of CD8^+^ T lymphocytes were induced by virus peptides and cells were analyzed on a four-color flow cytometer. Expression of CD107a, intracellular expression of interferon-γ (IFN-γ) and tumor necrosis factor-α (TNF-α), and cell proliferation assay were analyzed by using carboxyl fluorescein diacetate succinimidyl ester (CFSE).

**Results:**

A total of 30 patients with cerebral infarction and 30 healthy volunteers with an average age 57 (range, 49 to 71) years, were evaluated. The PBMCs were separated from blood samples of both, patients with cerebral infarction 6 hours after onset of stroke and healthy volunteers. After stimulation with virus peptides, CD107a expression and intracellular production of IFN-γ and TNF-α was decreased in patients with cerebral infarction as compared to healthy volunteers (p < 0.01). Degranulation analysis reported decreased expression of CD107a + in patient group as compared to healthy group, p <0.01. A mild decrease in intracellular expression of IFN-γ and TNF-α was also shown in patients without stimulation of virus peptides (p < 0.05). However, proliferation of CD8+ T lymphocytes in patients with acute severe cerebral infarction was not decreased.

**Conclusions:**

The study results indicated that cytotoxic function of CD8+ T lymphocytes were suppressed in patients with acute severe cerebral infarction. This could possibly be associated with complicated infectious diseases and neuroprotective mechanism.

## Background

Cerebral infarction, a cerebrovascular disease, is the most frequent disease of the brain and the leading cause of mortality worldwide. Cerebral infarction is an ischemic stroke resulting in cell death due to lack of blood supply to the brain. Complications after ischemic stroke are common and related to poor prognosis [1]. Infection is the most frequently occurring complication in patients with acute cerebral infarction and is difficult to control [2,3]. Numerous immunohematologic abnormalities have been reported with infection-associated cerebral infarction [4].

The immune system is responsible for protection against infectious diseases. Some studies have confirmed that patients with alterations in immune function are at increased risk of infection or decreased immunity [4-6]. The CD8+ T lymphocytes (cells) and natural killer (NK) cells are components of the adaptive immunity and innate immunity, respectively and constitute cellular immunity for infections. Previous studies emphasized the role of NK cells than CD8+ T lymphocytes. It has also been reported that adaptive immunity was inferior to innate immunity in exerting the cytotoxic effect. However, recent immunological researches support the role of CD8+ T lymphocytes for the elimination of infectious pathogens from the body [7,8]. This effect against pathogens is exerted by secreting cytokines like interleukins (ILs), interferon-γ (IFN-γ), and tumor necrosis factor-α (TNF-α). The cytotoxic responses have shown beneficial effects in controlling various infections and tumors [7].

The incidence of infection as the most common complication as well leading cause of death is on the rise in patients with stroke [3,6]. Suppression of immune system after cerebral infarction increases susceptibility to infections and significantly affects survival of patients after stroke. Ischemic stroke involves both innate and adaptive immunity. However, adaptive immunity has no impact in the acute stage of stroke, but modulation of adaptive immunity can exert a protective effect [9].

The objective of the present study was to assess the changes in immune function after stroke and to analyze cytotoxic function of CD8+ T lymphocytes in peripheral blood of patients with acute severe cerebral infarction.

## Methods

### Patient selection

This study was conducted from January 2009 to January 2011. Patients with clinical evidence of acute severe cerebral infarction were eligible for enrolment in the study (n=30). Other inclusion criteria were age 40 – 80 years, radiographic evidence of acute cerebral infarction, patients hospitalized within 6 hours after the onset of stroke, a score of not less than 16 as per the National Institutes of Health Stroke Scale (NIHSS) on admission. The exclusion criteria were: if the onset was along with infectious and autoimmune diseases, patients who received thrombolytic therapy after the onset, patients receiving immune regulation therapy within 6 months before the onset, patients with history of cerebral stroke within 12 months before the onset, patients who had history of severe cerebral trauma or neurosurgery, seizures during the onset and at the time of admission, renal or hepatic insufficiency at admission, history of blood transfusion within 12 months before the onset, severe trauma or surgery within 2 weeks before the onset, and history of severe psychological disease. A group of healthy adult volunteers (n=30; age: 40-80 years) were also included in the study for comparison. All patients or their family members provided signed informed consent and the protocol was approved by The Committee of Medical Ethics, East Hospital, Tongji University.

### Antibodies and reagents

Only mouse anti-human antibodies (purchased from BD Biosciences, USA) were used in the study. This included allophycocyanin (APC) conjugated to CD3 (CD3-APC), peridinin chlorophyll protein (PerCP) conjugated to CD8 (CD8-PerCP), fluorescein isothiocyanate (FITC) conjugated to cluster of differentiation 107a (CD107a-FITC), and phycoerythrin (PE) conjugated to interferon-γ (IFN-γ-PE) and tumor necrosis factor-α (TNF-α-PE). Ficoll-Biocoll, a cell isolation liquid and phosphate-buffered saline (PBS) were used (Biochrom AG, Germany). Perm/Wash™ buffer was used in the analysis to serve as an antibody diluent and cell wash buffer (BD Biosciences, USA). Cytofix/Cytoperm™ solution (BD Biosciences, USA) was used to increase the permeability of cell membrane. BD GolgiStop™ (BD Biosciences, USA), was used as a protein transport inhibitor containing monensin and was required to promote cytokine accumulation in the Golgi complex. RPMI-1640 (Roswell Park Memorial Institute medium, Sigma, Germany) was used as the cell culture medium (CTM). Cells were cultured in RPMI-1640 supplemented with 20 mM HEPES ([4, (2-hydroxyethyl]-1-piperazineethanesulfonic acid), 2 mM glutamine, 1% penicillin/streptomycin (Sigma, Germany), and heat-inactivated 10% human AB serum (3H Biomedical AB, Sweden). CellTrace™ carboxyl fluorescein diacetate succinimidyl ester (CFSE) was used as a cell proliferation reagent (Invitrogen, USA). Trypan blue solution (0.4%) was used to assess cell proliferation and viability by dye exclusion method (GIBCO, UK). Recombinant human interleukin-2 (rhIL-2) was purchased from ProSpec (Rehovot, Israel) and CD28/CD49d costimulatory antibody from BD Biosciences (USA). Mixed virus peptide used in the study was CTL-CEF-Class I peptide pool “Plus” (CEF peptide) (Cell Technology Ltd, USA). CEF peptide was a pool of 32 peptides, with sequences derived from the human cytomegalovirus, Epstein-Barr virus, and influenza (flu) virus.

### Isolation of peripheral blood mononuclear cells

PBMCs were isolated from whole blood as the red cells could have interfered with the flow cytometer analysis. In our preliminary experiments, we observed the apoptotic ratio of isolated PBMCs with Ficoll-Biocoll method and with lysate method (data not shown). Annexin V Apoptosis Detection Kit was used to detect apoptosis. The viability of PBMCs obtained was always > 95% with Ficoll-Biocoll method and < 50% with Lysate method. Hence, Ficoll separation method was used in the study.

Peripheral venous blood (20 ml) samples were collected from patients with cerebral infarction 6 hours after the onset of stroke in a heparinized test tube. The entire blood sample was diluted to a final volume of 20 ml with PBS. This suspension was then poured into 7 ml Ficoll-Biocoll separating solution in a conical tube. After density gradient centrifugation (1500 rpm, 20°C, 30 minutes, without brake), peripheral blood mononuclear cells (PBMCs) were isolated. The isolated cells were collected carefully and washed with sterile PBS and re-suspended in RPMI-1640. Cell viability was determined by trypan blue staining and cells were counted; cell viability should always be > 95%. Final density of the cell in CTM was adjusted to 5-6 × 10^6^ cells/ml. PBMCs from healthy volunteers were isolated in the same way. Collection of blood from the patient population and healthy volunteers was carried out at Philipps-University Marburg, Germany.

### CD107a degranulation analysis

For the expression of CD107a, 20 μl of CD107a-FITC antibody was added into 80 μl of PBMCs suspension (2-3 × 10^6^ cells/ml). PBMCs suspension mixed with CD107a was dispensed in a flat-bottom 96-well plate. Into each well, 2 μl of CD28/CD49d costimulatory antibody was also added. Further, 100 μl of CEF peptides with concentrations of 64 μg/ml were added in the CEF-treated-control group, and the same amount of CTM in the negative control group. The culture was incubated for 60 minutes at 37°C in 5% CO_2_ incubator after which 0.5 μl BD GolgiStop containing monensin was added. The incubation was continued for 120 minutes. After washing, cells were stained with CD3-APC and CD8-PreCP antibodies and incubated for 30 minutes at 4°C in the dark. Cells were centrifuged, supernatant was discarded and resuspended in 130 μl of CTM. Cells were analyzed on the four-color flow cytometer (FACSCalibur®, CellQuest® software, Becton Dickinson). At least 50000 events (events refer to the number of particles recorded by flow cytometry) were collected per cell. In the lymphocyte gate, CD8+ T lymphocyte for CD107a expression was defined as CD3+/CD8+/CD107a.

### Intracellular IFN-γ and TNF-α analysis

For analysis of intracellular IFN-γ and TNF-α, 2 μl CD28/CD49d costimulatory antibody was added to 100 μl of PBMCs. This suspension was dispensed into flat-bottom 96-well plate. Further, 100 μl of CEF peptides with concentration of 64 μg/ml was added in the CEF-treated-control group, and the same amount of CTM in the negative control group. The culture was incubated for 60 minutes at 37°C in 5% CO_2_ incubator and 0.5 μl BD GolgiStop containing monensin was added. This was further added at an interval of 6 hours during the next 24-hour incubation. Cells were centrifuged and supernatant was discarded. After washing, cells were stained with CD3-APC and CD8-PerCP antibodies, and incubated at 4°C for 30 minutes in the dark. Cells were then fixed with the 100 μl of BD Cytofix/Cytoperm solution and incubated for 20 minutes at 4°C in the dark. After washing and centrifugation, cells were suspended in Perm/Wash buffer solution and 20 μl of IFN-γ-PE and 20 μl of TNF-α-PE antibodies were added and incubated for 30 minutes at 4°C in the dark. Cells were analyzed on the four-color cytometer (FACSCalibur®, CellQuest® software, Becton Dickinson). Data from at least 50000 events per cell were acquired. In the lymphocyte gate, IFN-γ positive CD8+ T lymphocyte was defined as CD3+/CD8+/IFN-γ + and TNF-α positive CD8+ T lymphocyte was defined as CD3+/CD8+/TNF-α + .

### Cell proliferation analysis

CFSE stock solution was prepared and added into PBMCs in culture media. The working concentration of CFSE used was 0.4 μM. The culture media was incubated in the dark for 10 minutes at 37°C. The freed CFSE was inactivated with ice on CTM. It was again centrifuged and washed and cells were resuspended in CTM. The cell density was set at 2-3 × 10^6^ cells/ml. Cell suspension (stained with CFSE) was then dispensed into a flat-bottom 96-well plate. In cell suspension, 2 μl of CD28/CD49d costimulatory antibody was added. Then 100 μl of CEF peptides with concentration of 64 μg/ml and rhIL-2 with final concentration of 40 IU/ml was added in the CEF-treated-control group, and the same amount of CTM was added in the negative control group. After 5 days of incubation at 37°C in 5% CO_2_ incubator, cells were centrifuged, supernatant was eliminated and CD3-APC and CD8PerCP antibodies were added and incubated for 30 minutes at 4°C in the dark. Cells were again centrifuged, supernatant was discarded, washing was repeated, and cells were resuspended in 130 μl of CTM. Cells were analyzed on the four-color flow cytometer (FACSCalibur®, CellQuest® software, Becton Dickinson). At least 50000 events were collected per cell. The percentage of proliferating cells was measured by the percentage of low CFSE cells in CD3+/CD8+ gate (in the upper left quadrant of each plot). The definition for low CFSE cells was defined according to the distribution of CFSE dye in baseline, which was measured in unstimulated cells. CFSE decrease was a result of dye dilution in each cell division.

### Statistical analysis

All values were expressed as mean ± standard deviation (SD). Continuous data were analyzed using the paired t-test. For statistical comparisons, a p value less than 0.05 was considered to be significant.

## Results

### Patient characteristics

The study included 30 patients (17 males and 13 females) with acute severe cerebral infarction and 30 healthy adult volunteers (17 males and 13 females). The mean (SD) age of the patients with cerebral infarction and the healthy volunteers was 56.9 ± 13.5 (range: 50 to 69) years and 57.1 ± 14.5 (range: 49 to 71) years, respectively. There was no significant difference between both groups for demographic characteristics such as age, gender and weight (Table 1).

**Table 1:** The demographics data of cerebral infarction patient group and healthy volunteer group

	**Patient group (N = 30)**	**Healthy volunteer group (N = 30)**	**p value**
Male: female	17:13	17:13	>0.05
Age, y (mean)	56.9 (50-69)	57.1 (49-71)	>0.05
Weight, kg (mean)	77 (65-90)	75 (63-90)	>0.05

### Changes in immune function

Results from CD107a degranulation, CFSE cell proliferation and intracellular IFN-γ and TNF-α analysis for patients with cerebral infarction and healthy volunteers are shown in Table 2. Statistical analysis shows that after stimulation of CD8+ T lymphocyte by CEF peptide, there were significant differences between the two groups (p < 0.01) for both CD107a expression on the cell surface and intracellular expression of IFN-γ and TNF-α. After activation of CD8+ T lymphocyte, expression of CD107a and production of pro-inflammatory cytokines in patients with cerebral infarction was decreased compared to healthy volunteers. However, there was no statistical difference in the degree of cell proliferation between the two groups (p > 0.05).

**Table 2:** **Parameters of cytotoxicity of CD8**^**+**^**T lymphocytes in cerebral infarction patient group and healthy volunteer group with or without stimulation by virus peptides**

	**Cerebral infarction patient group**		**Healthy volunteer group**	
	**Not stimulated**	**Stimulated**	**Not stimulated**	**Stimulated**
CD107a + (%)	0.9 ± 0.6	10.6 ± 3.7**	0.8 ± 0.4	15.6 ± 4.3
IFN-γ + (%)	3.0 ± 1.2 *	15.3 ± 4.3**	3.6 ± 1.7	20.0 ± 5.1
TNF-α + (%)	3.3 ± 1.4*	15.1 ± 4.4**	4.1 ± 2.1	19.3 ± 4.7
Low CFSE (%)	0.7 ± 0.6	9.0 ± 3.5	0.9 ± 0.5	10.1 ± 4.0

All values are mean ± SD; ** p < 0.01, *p < 0.05, comparing between cerebral infarction patient group and healthy volunteer group. CD107a: cluster of differentiation 107a; *CFSE* Carboxyl fluorescein diacetate succinimidyl ester, *IFN* Interferon, *TNF* tumor necrosis factor.

Comparing the negative control groups (it refers to the healthy volunteer group not stimulated by CEF peptide), it was found that the content of intracellular pro-inflammatory cytokines of CD8+ T lymphocyte in patients with cerebral infarction was lower as compared to healthy volunteers (p < 0.05). No other differences were found.

**Figure 1:**
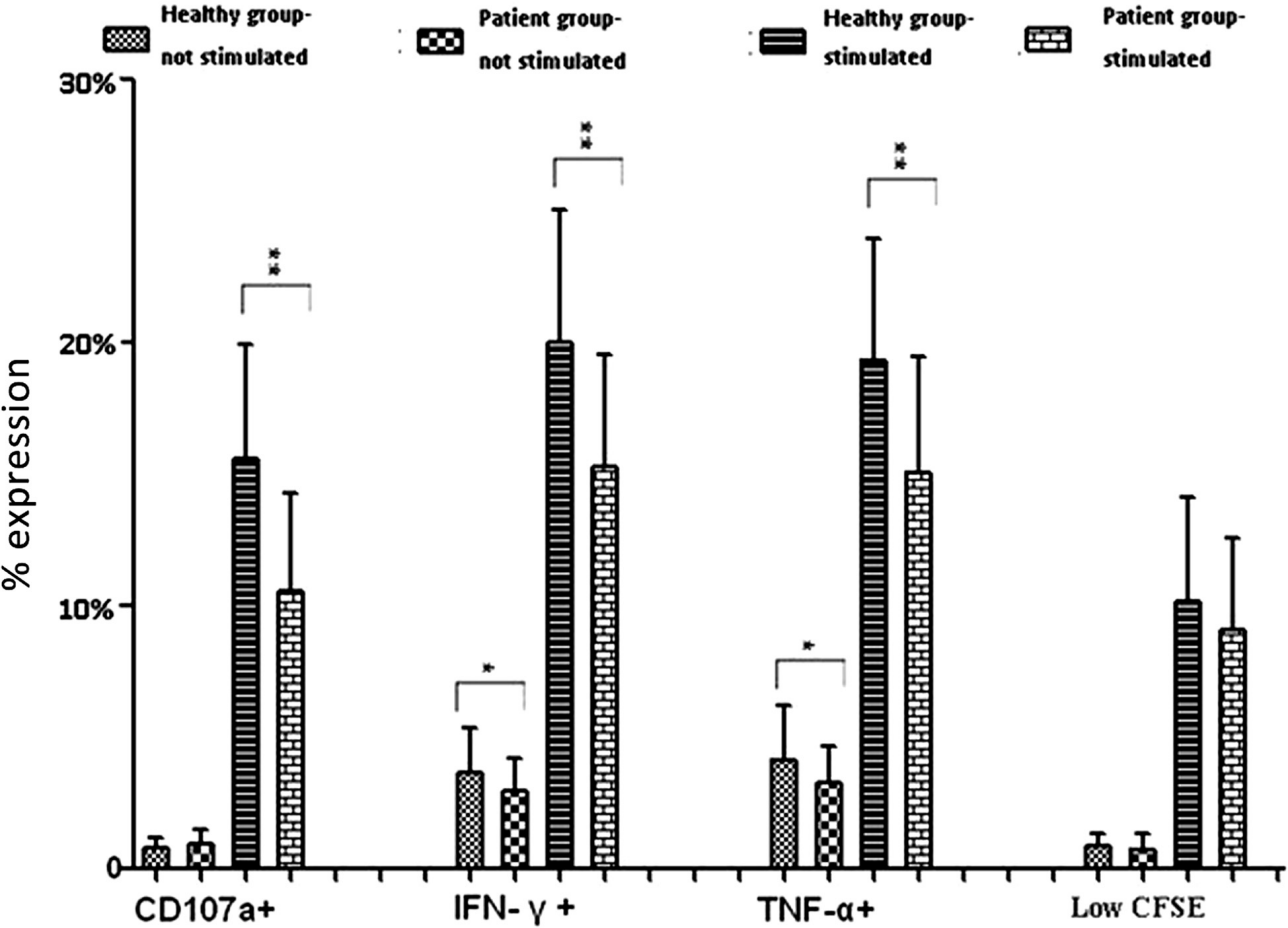
**Comparison of expression of CD107a, IFN-γ and TNF-α and CFSE dilution degree between patients and healthy group.** Expression of CD107a, IFN-γ and TNF-α, and CFSE dilution degree after CD8+ T lymphocytes stimulated and not stimulated by CEF peptide in vitro at different time points (CD107a: 3 h; IFN-γ and TNF-α: 24 h; CFSE dilution degree: 5d). Data is presented as mean ± SD. One-tailed t-test is used for analysis; ** p < 0.01; *p < 0.05. CD107a: cluster of differentiation 107a; CFSE: carboxyl fluorescein diacetate succinimidyl ester; IFN: Interferon; TNF: tumor necrosis factor.

The expression of CD107a, intracellular expression of IFN-γ and TNF-α, and cell proliferation between patient and healthy volunteer groups were assessed by CFSE (Figure 1). Cytokine expression (IFN-γ and TNF-α) and CD107a expression was significantly decreased in the patient group as compared to the healthy volunteer group, p <0.01.

### Degranulation analysis

**Figure 2:**
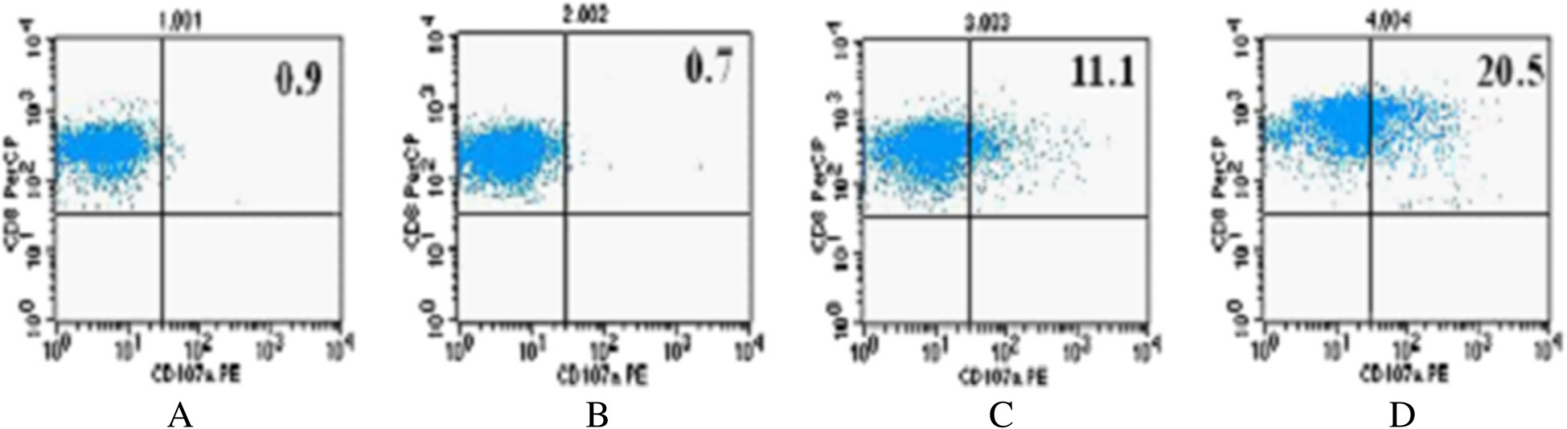
**Comparison of CD107a expression in patients with cerebral infarction and healthy volunteers with and without stimulation with CEF peptide.** Expression of CD107a between the two groups stimulated and not stimulated by CEF peptide in vitro. **A** is the contrast of cerebral infarction patient without stimulation; **B**, the comparison of healthy volunteer without stimulation; **C** shows the increase of CD107a + cells in cerebral infarction patients after stimulation with CEF peptide for 3 h; and **D** shows the increase of CD107a + cells in healthy volunteers after stimulation with CEF peptide for 3 h. CD107a: cluster of differentiation 107a.

Degranulation analysis of CD8+ T lymphocytes was performed to check for the expression of CD107a on the cell surface. Degranulation analysis conducted by comparing the percentage of CD107a + expression in patients with infarction and healthy volunteers is presented in Figure 2. After 3-hour stimulation of CD8+ T lymphocytes by CEF peptide *in vitro*, it was found that there was a decreased expression of CD107a + in patients as compared to healthy volunteers, p < 0.01.

### Intracellular expression of IFN-γ and TNF-α

**Figure 3:**
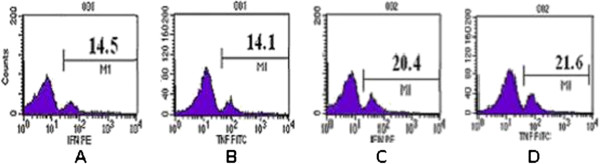
**Comparison of intracellular expression of IFN-γ and TNF-α in patients with cerebral infarction and healthy volunteers after stimulation with CEF peptide for 24 h *in vitro*.A** is the IFN-γ expression of cerebral infarction patient; **B**, the TNF-α expression of cerebral infarction patient; **C** shows the IFN-γ expression of healthy volunteer; and **D** shows TNF-α expression of healthy volunteer.

Contrasting images for intracellular expression of IFN-γ and TNF-α in cerebral infarction patients and healthy volunteers after 24-hour stimulation of CD8+ T lymphocytes by CEF peptide *in vitro* is shown in (Figure 3). Flow cytometry analysis revealed decline in concentration of IFN-γ and TNF-α in the patient group as compared to the healthy volunteer group.

### Cell proliferation analysis

Data for the percentage of proliferating cells after 5-day stimulation of CD8+ T lymphocytes by CEF peptide *in vitro* have been reported. There was no significant difference in the percentage of proliferating cells between patients with cerebral infarction and healthy volunteers on flow cytometry analysis.

## Discussion

Infection is considered as the cause as well as complication of stroke in patients with cerebral infarction. The incidence rate of infection in the acute stage of cerebral infarction is about 16% to 27% [2,3], this frequency rises in severe cases of infarction. This indicates the possibility of dysfunction of immune system. However, limited data is available on the function of CD8+ T lymphocytes. Our study found that the cytotoxic function of CD8+ T lymphocytes in the peripheral blood of patients with severe cerebral infarction was suppressed. This resulted in decrease rate of degranulated cells and pro-inflammatory cytokine production after stimulation by mixed virus peptides *in vitro*. However, cell proliferation was not affected. Before stimulation of CD8+ T cells with CEF peptides, there was a small difference (p < 0.05) between patients with cerebral infarction and healthy volunteers for intracellular pro-inflammatory cytokines of CD8+ T lymphocytes. One possibility was that severe stroke might possibly induce mild release of IFN-γand TNF-α from CD8+ lymphocytes, needs further investigation.

A study by Peterfalvi et al, reported that pro-inflammatory and cytotoxic responses of NK, NKT-like and Vdelta2 T cells become acutely deficient in ischemic stroke, which may contribute to an increased susceptibility to infections [10].

BD GolgiStop containing monensin was used in the current study with the objective to prevent the degradation of fluorescence on CD107a antibody and also to inhibit the transposition of intracellular cytokine from golgi body to the outside to limit the effect induced by cytokine release [11].

The CD8+ T lymphocytes are considered as the key effector cells in the adaptive immune response. The cytotoxic mechanism works mainly through pathway of degranulation and non-degranulation [12-14]. The former means that the cells will release cytotoxins containing perforin and various granzyme after activation, which results in direct paralysis of the target cell or apoptosis. However, the later indicates that the apoptosis of the target cell is induced through the production and release of cytokines such as IFN-γ and TNF-α [15]. Our study revealed that compared to healthy volunteers, the two pathways mentioned above were probably suppressed in patients with severe acute cerebral infarction, resulting in inhibition of cytotoxic function.

Cell proliferation is mainly responsible to enlarge the cytotoxic effects of CD8+ T lymphocytes, but the proliferation of CD8+ T lymphocytes in patients with cerebral infarction was not suppressed in our study. *In vitro* culturing of cells has major differences from those *in vivo* (as in patients with cerebral infarction), especially in terms of the cell conditions and time of proliferation.

The suppression of the cytotoxic function of CD8+ T lymphocytes in patients with severe cerebral infarction lowers the patient’s resistance to infection, but it may also have some protective effect. Recent animal studies have proven that CD8+ T lymphocytes induce neurotoxic effects in the early stage of acute cerebral ischemia. In a study with gene knock-out of CD8 + T lymphocytes by Yilmaz et al., the size of cerebral infarction in mice was distinctly reduced [16]. Therefore, the cytotoxic function of CD8+ T lymphocytes may be attributed to a self-protective mechanism [17].

In the current study, it was observed that without stimulation, patients with cerebral infarction had mild decline in intracellular pro-inflammatory cytokines of CD8+ T lymphocytes than the healthy volunteers. This indicated that severe cerebral infarction itself mildly induced CD8+ T lymphocytes to release IFN-γ and TNF-α. Further research in this area will elucidate the role of CD8+ T lymphocytes. It is worth noting that an accumulating body of evidence from experimental studies support a definite neurotoxic role of CD8+ T lymphocytes [16-18]. So, inhibiting the cytotoxic function of CD8+ T lymphocytes in acute severe cerebral infarction was a mechanism of self-neuroprotection. From a clinical viewpoint, the balance between activating and inhibiting the cytotoxic function of CD8+ T lymphocytes requires further investigation.

## Conclusions

Findings from our study show that the cytotoxic function of CD8+ T lymphocytes in patients with acute severe cerebral infarction was suppressed. These results will help in identifying the reasons of high infection rate and the difficulty in controlling the infection in patients with cerebral infarction. This may be attributed to the mechanism of self-neuroprotection.

## Abbreviations

APC: Allophycocyanin; CD107a: Cluster of differentiation 107a; CFSE: Carboxyl fluorescein diacetate succinimidyl ester; CTM: Cell culture medium; FITC: Fluorescein isothiocyanate; IFN: Interferon; IL: Interleukin; NK: Natural killer; PBS: Phosphate-buffered saline; PBMCs: Peripheral blood mononuclear cells; PE: Phycoerythrin; PerCP: Peridinin chlorophyll protein; TNF: Tumor necrosis factor.

## Competing interests

The authors declare that they have no competing interests.

## Authors’ contributions

GL carried out flow cytometry analysis and drafted the manuscript. XW carried out the design of study and participated in manuscript preparation. L-hH and X-yX carried out patient selection. YW carried out the isolation of peripheral blood mononuclear cells and statistical analysis. J-jH and XG performed the sample collection. All authors read and approved the final manuscript.

## Authors’ information

GL holds a qualification of MD and currently working as a Vice-Chairman in Department of Neurology, East Hospital, Tongji University. He is mainly engaged in immunology research in cerebrovascular disease. XW is a professor (MD) in Fudan University and Vice-President in Zhongshan Hospital, engaged in epilepsy and cerebral vascular diseases immunology and the neuroendocrine study. All others authors hold a qualification of MD.
